# PlasPi TDM: Augmentation of a low-cost camera platform for advanced underwater physical-ecological observations

**DOI:** 10.1016/j.ohx.2023.e00470

**Published:** 2023-08-28

**Authors:** Coffi Gérard Franck Zinzindohoué, Timm Schoening, Estanislau Baptista Lima, Björn Fiedler

**Affiliations:** a Universidade Técnica do Atlântico (UTA) – Instituto de Engenharias e Ciências do Mar (ISECMAR), Mindelo, São Vicente, Cabo Verde; b West African Science Service Centre on Climate Change and Adapted Land Use (WASCAL), Mindelo, São Vicente, Cabo Verde; c GEOMAR Helmholtz Centre for Ocean Research Kiel, Germany

**Keywords:** PlasPi TDM, Marine observations, Citizen science, Physical oceanography, Underwater imaging

## Abstract

Marine ecosystem dynamics in the context of climate change is a growing scientific, political and social concern requiring regular monitoring through appropriate observational technologies and studies. Thus, a wide range of tools comprising chemical, biogeochemical, physical, and biological sensors, as well as other platforms exists for marine monitoring. However, their high acquisition and maintenance costs are often a major obstacle, especially in low-income developing countries. We designed an advanced low-cost synoptic marine ecosystem observation system that operates at relatively high temporal frequencies, named PlasPi TDM. This instrument is an improved version of the camera system (PlasPI marine cameras) developed in 2020 by Autun Purser from the Alfred Wegener Institute Helmholtz Center for Polar and Marine Research (Germany), and collaborators. It incorporates several innovative developments such as multispectral (records the spectrum of any object photographed), temperature and pressure sensors. The PlasPi TDM operates to a depth of 200 m. The various field deployments demonstrate the operational capability of the PlasPi TDM for different applications and illustrate its considerable potential for in-situ observations and marine surveillance in Africa. This device is intended as an open-source project and its continued development is encouraged for a more integrated, sustainable and low-cost ocean observing system.

## Graphical abstract

**Figure:**
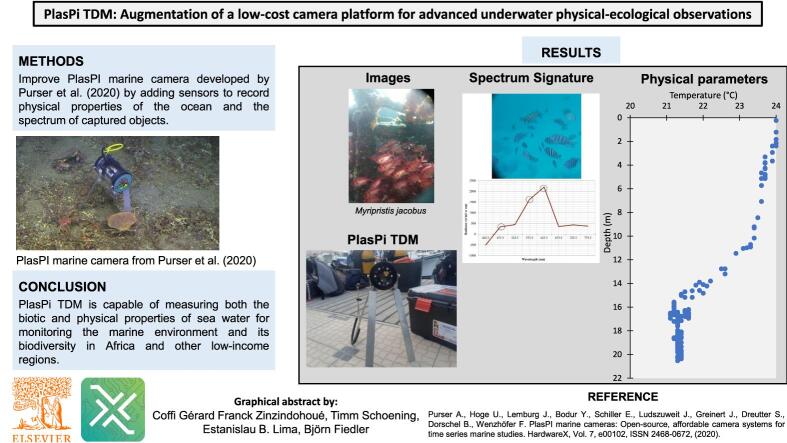

## Specifications table

**Table:** 

Hardware name	*PlasPi TDM*
Subject area	● Environmental, Planetary and Agricultural Sciences
Hardware type	● Imaging tools Field measurements and sensors
Open-Source License	*CERN Open Hardware Licence v1.2*
Cost of Hardware	PlasPi TDM: from 300 to 1300 Euro depending on the complementary sensors
Source File Repository	https://doi.org/10.5281/zenodo.7539553

## Hardware in context

Covering over 70% of the Earth’s surface, the oceans supply the entire world with regulatory, supporting, cultural and provisioning services [1]. The oceans are therefore an essential life support system (source of food, energy, transport, etc.) and the main biodiversity reservoir within the Earth’s system. However, the marine ecosystem is increasingly subject to many direct human pressures such as overexploitation, eutrophication, pollution, and invasive species [2], [3], [4], [5]. Moreover, pressure from technological advances, population expansion, and the global impacts of climate change [6] are accelerating marine environmental change. Contemporary climate change alters marine biogeochemical conditions, threatens the availability of ocean-derived services, creates novel oceanic conditions and undermines the regulatory potential of marine ecosystems [7]. Johnson & Watson (2021, [7]) revealed that climate-related stressors will drive major changes in up to 87% of the global oceans, and 97% of the largest MPAs, resulting in novel conditions that may influence species migration, extinctions, and invasions. According to these authors, it is imperative to understand and accurately predict contemporary complex oceanographic changes to effectively inform ocean-climate policy aimed at mitigating, avoiding, and/or adapting to anticipated changes. Thus, understanding when, where and how oceanographic changes will occur is essential to inform the sustainable use and management of ocean resources [8]. However, to achieve this, statistical prediction models are not sufficient and should be supplemented by observational techniques, preferably affordable low-cost ones.

In recent years, several oceanographic research projects have been carried out globally [9]. The challenges encountered during these expeditions have resulted in significant advancements in the design and construction of observational tools or instruments. These include a variety of underwater observation platforms, such as remotely operated vehicles (ROVs), baited remote underwater video (RUV) systems, autonomous underwater vehicles (AUVs), and seabed observation systems [10], developed to access and explore distant environments [11], [12] and overcome the challenges of monitoring marine environmental changes [13].

However, despite these advances, we observe the widening of the North-South divide in the development and use of technology and instruments in oceanographic research, particularly in Africa. Hence, one of the major hindrances to oceanographic research in Africa is the high cost of observational and underwater research equipment, which many countries, with minimal resources, cannot afford today. To overcome these limitations and help narrow the technological gap, cheaper innovative alternatives have emerged in recent years [14], [15]. These low-cost instruments are developed for specific purposes in marine research and differ according to the specific technologies used. For instance, low-cost sensors and probes developed by Marcelli et al., measure marine temperature, conductivity, chlorophyll and Chromophoric Dissolved Organic Matter fluorescence [15]. Lockridge et al., [16] developed a low-cost, robust, user-friendly probe, built on Arduino Mega 2560 (Mega) and Arduino Uno (Uno) platforms for coastal applications. This platform allows for the internal logging of several parameters, including conductivity, temperature and is equipped with a GPS positioning. The probe was used on a Lagrangian surface drift to record temperature, salinity and positional measurements. Duraibabu et al., [17] designed the first optical fibre-based point sensors that combine pressure (depth) and temperature sensors for marine and freshwater applications to monitor water column changes relative to depth [17].

Other instruments developed for advanced oceanographic observations, combining physical water measurement and underwater imaging technologies include the GUARD1 system, a versatile instrument that can be installed on various platforms, operating autonomously with programmable and adaptable features, designed by Marini et al., (2015) [47]. This low-cost, stand-alone instrument is suitable for underwater image acquisition and recognition of gelatinous zooplankton using dedicated algorithms. While the GUARD1 excels in jellyfish recognition with onboard data processing capabilities, it cannot gather critical environmental information along with multispectral data. Similarly, Dominguez-Carrió et al., [18] designed the Azor Drift-Cam, a new low-cost high-resolution video camera system for the rapid assessment of deep benthic habitats, but, the Azor Drift-Cam's cost and deployment requirements pose challenges, as it requires the use of a crane due to its size and weight.

In addition to visual imaging techniques, the development of spectral imaging devices mounted on Unmanned Aerial Vehicles or aircraft has evolved in recent years, largely for biodiversity detection, monitoring, and assessment [19], [20], [21], [22]. Jusoff [19] used airborne hyperspectral imaging to identify and map individual mangrove species in Port Klang and determined the wavelength regions that defined the inherent spectral characteristics of mangrove species. Schoonmaker et al., [20] also developed an airborne spectral imaging system consisting of a low-cost four-band ACT multispectral (MANTIS-3) system to detect, track and monitor marine mammal (whale) populations in St. Lawrence Seaway. Similarly, Lopez et al., [21], studied automated real-time detection of Great White Shark dummy targets using a multispectral imager. While Spectral imaging devices mounted on UAVs or aircraft exteriors have shown promise in biodiversity detection and monitoring [19], [20], [21], [22], they primarily provide aerial perspectives and may not capture detailed information about underwater conditions.

All of these technologies have significantly contributed to our scientific understanding of the seas and oceans. However, to address the need for ecological observations along the African coast and given the financial limitations associated with oceanographic research in this region, we developed the PlasPi TDM, an innovative cost-effective solution that offers versatility and a combination of sensors to enhance the broader framework of seabed and pelagic monitoring networks. With its ability to capture time-lapse images, collect environmental data, and integrate multispectral sensors, this new device provides valuable insights into marine ecosystems, supporting research and conservation efforts.

### Conceptual framework of the PlasPi TDM and advantages for coastal communities

**Fig. 1:**
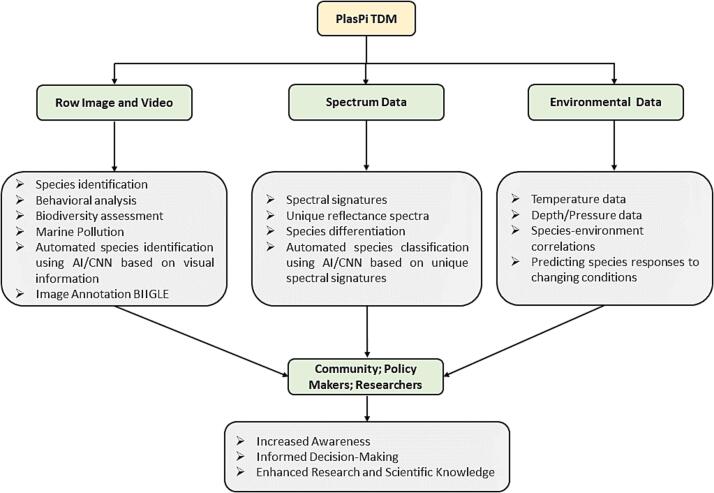
Conceptual framework of the PlasPi TDM.

Motivated by the above developments, we aimed to design and build an upgraded and advanced version of the low-cost PlasPi marine camera system developed by Purser et al., [23] and adapt it to oceanographic conditions in Africa’s marine environment. The goal of this system was to overcome the current challenges hindering oceanographic research in Africa, primarily the high cost of monitoring sensors for measuring the ocean’s physical and biological properties of water (complemented by imaging data acquisition). This the PlasPi TDM (Temperature-Depth-Multispectral), was built around the Raspberry Pi microcontroller and integrates a multispectral sensor, a temperature sensor, and a pressure/depth sensor. Unlike its predecessor, which was designed mainly to take pictures and record videos, the new device provides a comprehensive suite of tools to gather essential oceanographic data. This novel conceptual framework offers immense advantages for coastal communities in their efforts to study, monitor, conserve and manage the marine environment (Fig. 1).

The row images captured by the PlasPi TDM are integral to marine environmental monitoring and serve as visual documentation for identifying organisms, objects, and habitats. Analyzing these images provides additional insights into species composition, biodiversity, and changes in distribution, supplementing the results of other existing methods. Furthermore, the images offer valuable information on ecological interactions, behavioural patterns, and physical characteristics of organisms. The PlasPi TDM's multispectral sensor further enhances its capabilities by collecting data across various light wavelengths, enabling species identification, and classification, based on unique spectral signatures. The PlasPi TDM's temperature sensor and pressure/depth sensor play key roles in studying marine ecosystems. The temperature sensor measures variations in water temperature over time and depth, providing insights into oceanographic processes and identifying areas of thermal stress that affect marine organisms. On the other hand, the pressure/depth sensor provides valuable information about water column characteristics, such as vertical mixing, water mass properties, and depth profiles. These data are essential for understanding ocean dynamics, studying vertical migrations of organisms, and assessing the vertical distribution of marine life.

The PlasPi TDM, as an open-source tool, could empower African countries as well as other low-income countries in the Global South to effectively monitor their marine and coastal ecosystems. The data obtained will guide policies for sustainable observation and management. By integrating and analyzing the collected data, a comprehensive understanding of marine ecosystems, biodiversity, physical properties, and environmental changes will be achieved. This supports research, conservation, and informed decision-making for the sustainable management of coastal and oceanic resources.

## Hardware description

The integration of temperature/depth and multispectral sensors in the PlasPi TDM represents a significant advancement that enables comprehensive data collection and analysis, providing valuable insights into the physical properties of the marine environment and the spectral characteristics of encountered organisms. These capabilities are crucial for developing ecological indicators to assess and monitor ecosystem health and functioning, thereby contributing to a deeper understanding of ecological processes and facilitating the implementation of effective conservation and management strategies [29].

The PlasPi TDM also has significant potential in the growing field of AI/CNN-based protocols for automated identification, classification, and tracking of individuals of different species. As Naidu et al., (2022) [30] emphasized, advancements in artificial intelligence and computer vision have opened up new possibilities for efficient and accurate species identification and tracking in underwater environments. By combining image and video capture capabilities with a multispectral sensor, the PlasPi TDM provides a comprehensive dataset that includes visual information and spectral signatures of organisms. Species have differences in reflectance spectra due to their unique biophysical and biochemical properties [31], [32]. This could enable the training and improvement of AI/CNN algorithms, leading to automated species identification and classification based on unique spectral characteristics. Furthermore, the integration of a temperature/depth sensor adds another dimension to the potential applications of AI/CNN-based protocols. In the absence of auxiliary sensor platforms, on-board capturing of temperature data along with visual and spectral information, researchers can correlate environmental variables with species behaviour and distribution patterns, improving our ability to predict species' responses to changing environmental conditions.

The PlasPi TDM is designed to be easily transportable and deployed due to its lightweight. It is also a very versatile instrument that can operate in the open ocean and coastal waters in the absence of expensive platforms, considering energy constraints and operational depths. When the device is set to record a full range of data from all sensors at a high-resolution time scale, it is important to note that the duration of data collection is limited by energy constraints. To optimize power usage and extend deployment time, it is recommended to configure the device to capture data selectively or adjust the time intervals between captures based on specific research objectives and available power resources. This allows for a balance between data collection and energy efficiency, ensuring the device's suitability for long-term deployment and continuous observation. Additionally, it is important to note that the PlasPi TDM has specific operational depth limits, allowing for reliable data collection and observation at depths up to 200 m. The device is capable of capturing image resolution of 8 Megapixels and recording videos with a resolution of 640x480 pixels at brief time intervals and storing them efficiently as long as there is sufficient storage space. Additional characteristics include:

● System flexibility; the device is easy to set up and use without sophisticated technical expertise, and the components replacement process is straightforward.
● The measurement of the spectrum reflectance is done across 8 wavelengths in the visible spectrum, ranging from approximately 425––775 nm.
● Accurate measurements of temperature and pressure values, respectively, with an accuracy of ± 0.1 °C and 0.2 mbar in the water column.

## Design files

The fundamental design of the PlasPi TDM shares similarities with the PlasPI marine camera. However, due to the extended payloads via the addition of the temperature, pressure, and multispectral sensors, certain components, including the front and back-end cap and the front-end cap opening, have undergone modifications. The wiring diagram has also been significantly revised to reflect these changes. The remaining components of the camera system remain identical to the PlasPI marine system Camera described by Purser et al., 2020 [23]. Additionally, all the relevant design files of the PlasPi TDM can be accessed through the online repository, using the link provided in the table below.

**Fig. 2:**
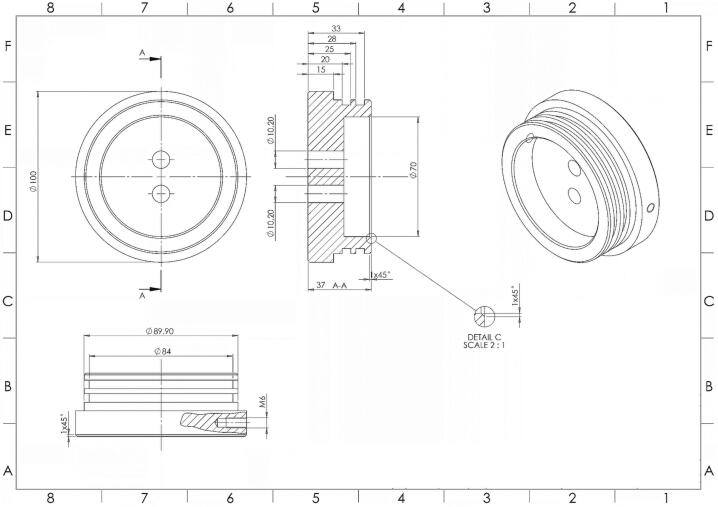
Schematic of the Back-End Cap of the PlasPi TDM (https://doi.org/10.5281/zenodo.7527461).

**Fig. 3:**
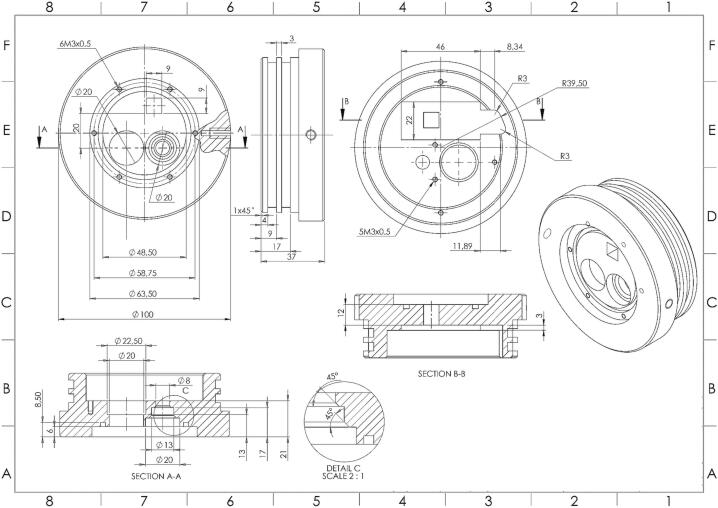
Schematic of the Front-End Cap of the PlasPi TDM (https://doi.org/10.5281/zenodo.7527487).

**Fig. 4:**
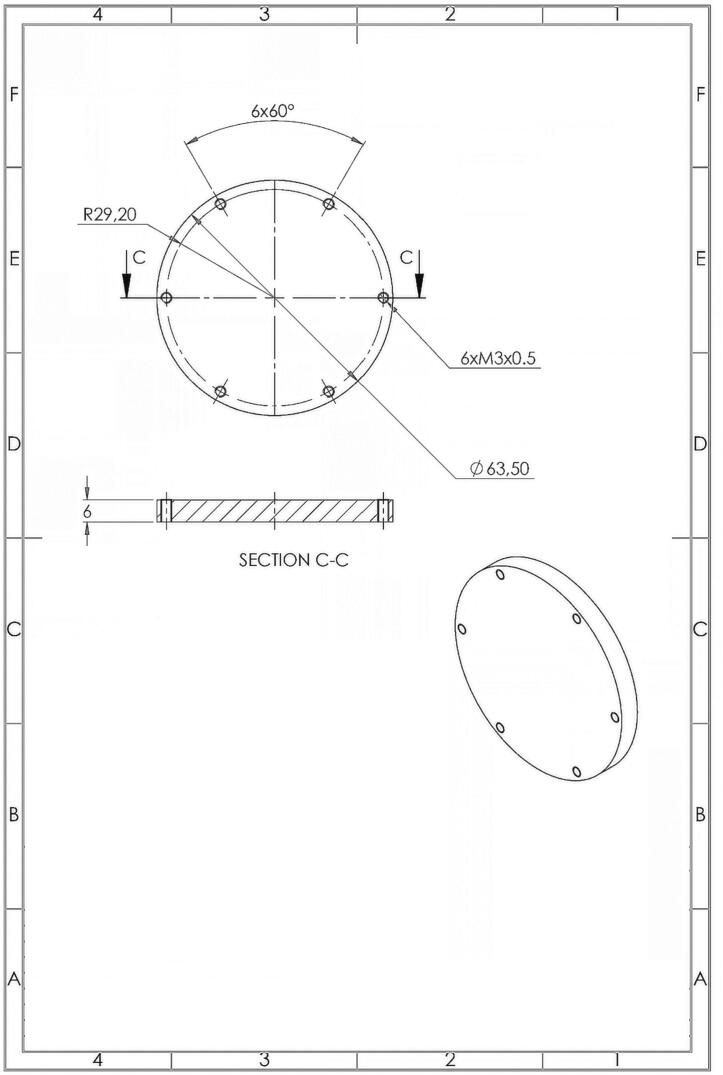
Schematic of the Front plexiglass of the PlasPi TDM (https://doi.org/10.5281/zenodo.7527490).

### Design files summary

**Table:** 

Design file name	File type	Open-source license	Location of the file
*Back-End Cap of the PlasPi TDM*	Fig. 2	*CERN OHL*	*10.5281/zenodo.7527461*
*Front-End Cap of the PlasPi TDM*	Fig. 3	*CERN OHL*	*10.5281/zenodo.7527487*
*Front plexiglass of the PlasPi TDM*	Fig. 4	*CERN OHL*	*10.5281/zenodo.7527490*
*Exploded view of the PlasPi TDM*	Fig. 6	*CERN OHL*	*https://doi.org/10.5281/zenodo.7527504*

## Bill of materials

**Table:** 

Designator	Component	Number	Cost per unit -Euro	Total cost - Euro	Source of materials	Material type
*1*	*PlasPI marine camera.*	*1*	*200*	*200*	*https://www.sciencedirect.com/science/article/pii/S2468067218300919*	*Non-Specific*
*2*	*Bar30 High-Resolution 300 m Depth/Pressure Sensor*	1	*98.77*	*98.77*	*https://bluerov-solutions.com/produkt/bar30-high-resolution-300m-depth-pressure-sensor/*	*Other*
*3*	*Celsius Fast-Response, ±0.1 °C Temperature Sensor (I2C)*	1	*78.54*	*78.54*	*https://bluerov-solutions.com/produkt/celsius-fast-response-%c2%b10-1c-temperature-sensor-i2c/*	*Other*
*4*	*I^2^C bus Splitter*	1	*15.59*	*15.59*	*https://bluerov-solutions.com/produkt/i2c-bus-splitter/*	Other
*5*	*PixelSensor OEM VIS-8-UVIR*	1	*878.62*	*878.62*	*https://opticalfiltershop.com/shop/sensing-and-imaging/pixelsensor/pixelsensor-evaluation-kit-vis/*	Other

### The PlasPi marine camera

The PlasPi marine camera is an open-source instrument [23], applicable in various scientific fields, including environmental, planetary, and agricultural sciences. It is a shallow water (150 m depth) camera system with a plastic pressure housing [23] for taking photos and recording videos.

### Temperature and depth/pressure sensors

The temperature sensor used as a follow-up extension to the PlasPI marine camera consists of the BlueRobotics Celsius Fast-Response [24] with an accuracy of ± 0.1 °C and communicates via I2C. Whereas, the BlueRobotics Bar30 High-Resolution [25] 300 m Depth was used for the pressure sensor. Having a resolution of 0.2 mbar and a depth measurement resolution of 2 mm in the water column, it also communicates over I2C.

### I2C bus splitter

The I2C is a bi-directional, synchronous serial communication interface which uses a standard clock signal to synchronize data transfer between devices. Each sensor is connected to the I2C bus splitter via its own I2C address. The I2C has two lines or pins: the SCL_I2C, which is the clock line to synchronize the transmission, and the SDA_I2C, which represents the data line through which data bits are sent or received by the interface. In this project, an I2C bus splitter was used to allow for the connection of the temperature and pressure sensors by sharing the same bus and avoiding any communication problems.

### PixelSensor OEM VIS-8-UVIR

Designed by Ocean Insight company, the PixelSensor OEM (Original Equipment Manufacturing) VIS-8-UVIR (Visible Spectrum-8-Ultraviolet Infrared) is a small and compact spectrometer measuring 8 wavelengths in the visible spectrum covering the range from approximately 425 to 775 nm [26].

## Build instructions

The different stages of the construction of the PlasPI marine camera have already been broadly discussed [23]. Here will focus on the technical integrations of the new auxiliary TDM sensors. We also described the design of the main components of the PlasPI marine camera housing, namely the back-end cap, the front-end cap and the plexiglass front that have been modified to accommodate the new auxiliary sensors.

### PlasPi TDM back-end cap

Fig. 2 shows the new PlasPi TDM back-end cap. The back-end cap is a machined and milled Polyoxymethylene (POM) component with a diameter of 100 mm. The back-end cap has been modified by creating two 10.2 mm diameter holes in the middle of the end cap to accommodate the Temperature and Pressure sensors. Two holes are also drilled on the sides of the end cap to allow later connection to the mounting frame [23]. Two O-ring rubber and silicone greases enable sealing when placed in the two grooves inside the back-end cap set in a piston-type mounting. The sensors are inserted through the holes from the outside to the inside of the back-end cap. An O-ring and silicon grease are used to ensure a tight seal. Then a nut is placed on the inside to secure each sensor.

### PlasPi TDM front-end cap

The original design of the front-end cap consisted of two square acrylic plastics covering the camera and LED ports individually. Two ports are cut out of the POM to allow the LED and camera to illuminate and image the water column. The acrylic plastic port windows are also required to cover these ingresses [23]. Our prototype features a different front-end cap design (Fig. 3). Two holes are drilled on the sides of the front-end cap for later connection to the mounting frame. Additionally, three holes are drilled into the POM (Polyoxymethylene) component to accommodate the camera, the LED, and the multispectral sensor to image, illuminate and record the reflectance spectrum of objects in the water column, respectively.

### Plexiglass front end-cap opening

The front cover (Fig. 4) is circular in contrast to the square shape of the original design and made of plexiglass, providing coverage for the entire opening of the front-end cap. The simplified design of a single circular front cover streamlines manufacturing and assembly processes, potentially reducing associated costs and time requirements. Additionally, the circular shape of the front cover demonstrates efficient material usage by eliminating unnecessary overlaps or gaps. This optimized material utilization can help reduce waste during production, contributing to cost efficiency. To ensure proper sealing, two silicone-grease O-ring type rubber seals are positioned in the grooves inside the back-end cap. Additionally, a rubber seal and silicone grease are applied to the front of the front-end cap. All seals are assembled in a piston-type arrangement with six holes available for M3 screws.

### Exploded view of the PlasPi TDM

**Fig. 5:**
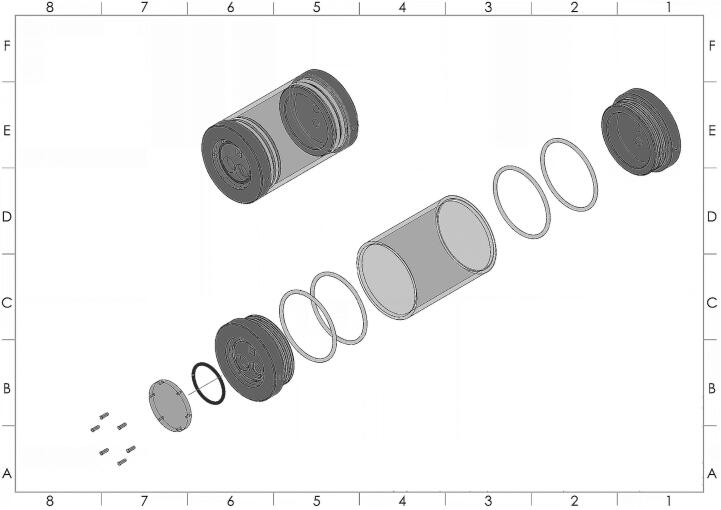
Exploded view of the various assembled parts of the PlasPi TDM (https://doi.org/10.5281/zenodo.7527504).

The exploded view of the PlasPi TDM (Fig. 5) showcases the individual components and their spatial arrangement within the device. This visual representation provides a clear depiction of how the different parts are assembled and interact. It gives a better understanding of the internal structure and layout of the PlasPi TDM, enhancing the comprehension of its functionality and design. It allows for a detailed examination of the relationships between components, revealing the interconnectedness and integration of these elements in the overall system.

**Fig. 6:**
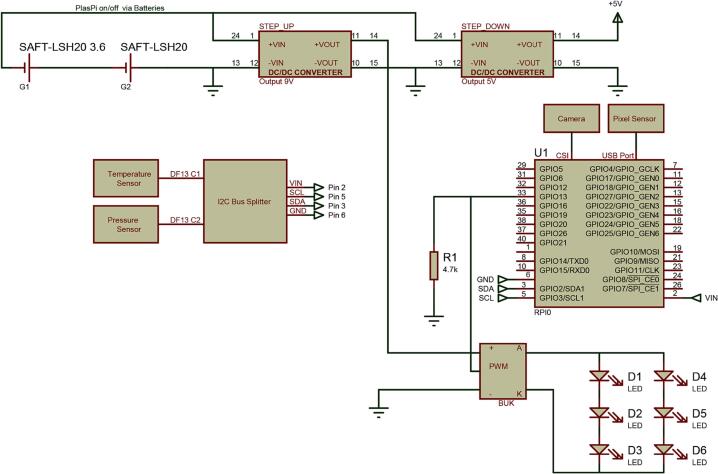
Wiring diagram of the PlasPi TDM.

### Wiring of the auxiliary sensors to the microcontroller (raspberry Pi zero w)

**Fig. 7:**
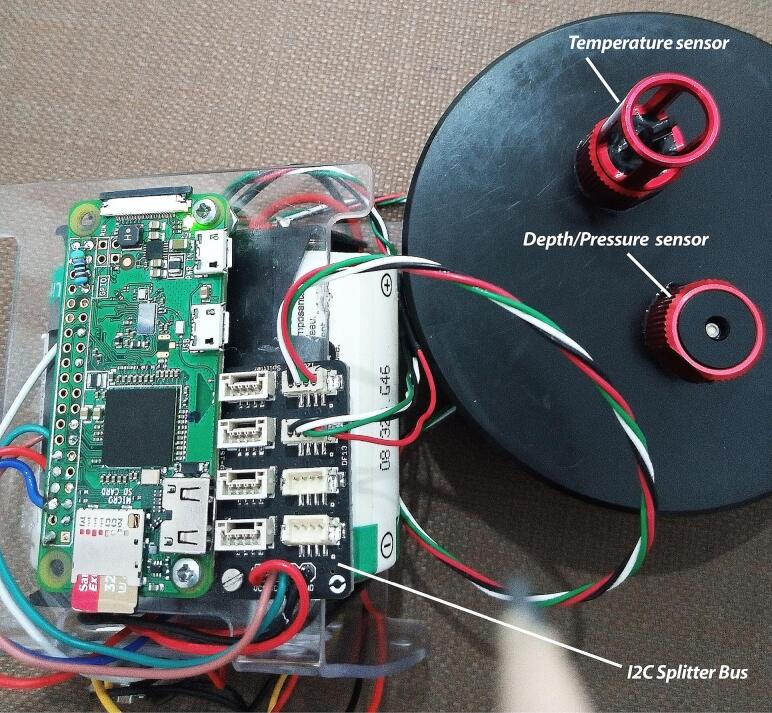
Temperature and pressure sensor connected to the I2C Splitter Bus.

The multispectral sensor is connected to a Raspberry via a USB Port. The temperature and pressure sensors are plugged into the i2c bus splitter via SCL, SDA, VIN, and GND (Fig. 7), which in turn is connected to the Raspberry's I2C bus using two pins (GPIO 2/Pin 3 and GPIO 3/Pin 5). Each sensor is connected via its own I2C address. The SCL, SDA, VIN, and GND pins of the I2C bus splitter are connected to the SCL (GPIO 3/Pin 5), SDA (GPIO 2/Pin 3), VIN (Pin 2), and GND (Pin 6) of the Raspberry Pi, respectively. Fig. 6 shows the complete wiring diagram of the PlasPi TDM.

## Operation instructions

The operation instructions for use are almost similar to those described by Purser et al., [23]. However, due to the changes made to our prototype, additional instructions are highlighted covering the pre-deployment and benthic deployment phases of the device.

### Pre-deployment

#### Hydrostatic pressure testing of the equipment

In the marine environment, the maximum safe operating depth of the device’s enclosure depends on the material of construction and its assembly. Therefore, before mounting the electronics onto the enclosure, a hydrostatic test must be performed. The hydrostatic test consists of subjecting the enclosure to different pressure levels to ensure that it functions correctly. The enclosure may also be tested in a tank to detect leaks or bubbles, Preferably, and if possible, testing should also be done at sea.

#### Connecting the auxiliary sensors

The LED, camera and multispectral sensor must first be mounted in the front-end cap. Then the mounting frame (refer to Purser et al., [23]) containing all electronic components and the power supply should be attached to the front end. The temperature and pressure sensors should be mounted in the rear end cap, which in turn will be sealed to the cylinder with the use of silicone grease O-rings. This process will allow the temperature and pressure sensors to be easily connected later to the i2c splitter bus located at the mounting frame.

#### Bench test

Before deployment in the natural environment, it is important to conduct a bench test, a functional test in which the device is physically tested before actual deployment. This allows for recreating the scenarios in which the device will be used to analyze its behaviour, improve the camera parameters for photography and video recording, and check that all the data (temperature, pressure, spectral) are recorded as desired.

#### Security measures before closing the Housing

Since the PlasPi TDM is an aquatic device, it is recommended that silica packets are placed in the housing to keep the components inside the housing dry. This is a preventive measure in case the device is not fully sealed by the O rings and silicone.

### Benthic deployment

For benthic deployment, the PlasPi TDM can be attached to landers, moorings or coring devices. However, it is advisable to add an approximate weight of about 8 – 10 kg to the attachment gear to prevent drifting with ocean currents.

### After recovery of the device

Depending on the depth at which the device is deployed, excessive pressure may build up in the tube. In this case, it is recommended to open the device carefully to prevent damage to the enclosure.

## Validation and characterization

### Hydrostatic pressure test

The housing of the PlasPi TDM, made of durable polycarbonate material, is designed to withstand the demanding conditions of underwater environments. With a diameter of 90 cm, a length of 130 cm, and a width of 100 cm, the housing provides ample space for accommodating the various components of the device. The inner edges of each tube end are slightly filed, allowing for easier access to the back and front end of the housing during maintenance and assembly processes.

**Fig. 8:**
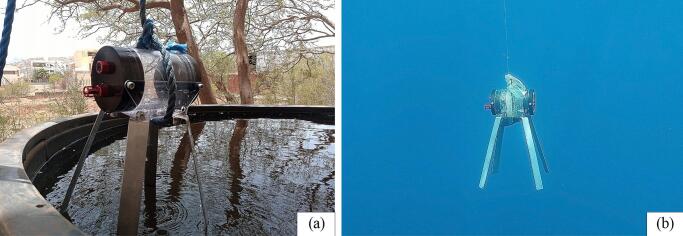
(a) The housing being taken out of the tank after the hydrostatic pressure test, and (b) the housing in the water column during the hydrostatic pressure test carried out at sea.

The housing's operational depth range has been tested and validated. In initial tests, the housing was placed in a 4200 L tank (Fig. 8 (a)) to ensure its integrity and check for any leaks or bubbles. Subsequently, the housing underwent rigorous hydrostatic pressure testing at sea (Fig. 8 (b)). The pressure was steadily increased to a maximum depth of 200 m, surpassing the intended 150 m depth for the study. Each test was conducted for 45 min to simulate real-world deployment conditions.

The results of the housing's performance evaluation were highly satisfactory. After dismantling the housing from the tank, it was thoroughly checked, and no water or leakage was observed. During the hydrostatic pressure test at sea, the housing successfully withstood pressure equivalent to 20 bars (at a depth of 200 m) for the entire 45-minute duration without implosion or water leakage. These dimensions and successful performance tests demonstrate the housing's robustness and reliability, ensuring the safe and effective deployment of the PlasPi TDM in underwater environments. This increases confidence and assurance in the use of the device to conduct advanced underwater ecology observations and data collection at depths of up to 200 m.

### Bench test

**Fig. 9:**
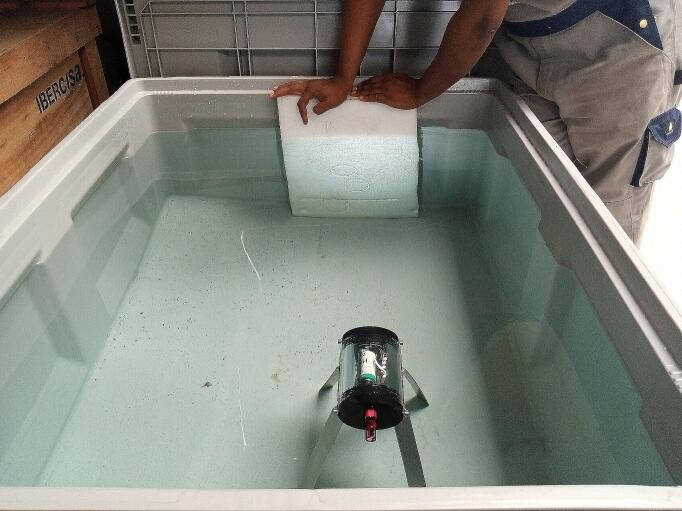
Bench test of the PlasPi TDM.

The waterproofness of the device was once again tested, but this time with the whole electronic part inside the housing (Fig. 9). No water ingress was observed. During this test, several data acquisition modes were used: Photo and physical data collection mode, photo + video + physical data collection mode, and video + physical data collection mode. Each mode was tested for 45 min. The various data obtained from these tests are consistent with those expected.

### Field test validation

The field validation was carried out at four different locations on the island of São Vicente in Cabo Verde. These include São Macario, an 80 m long shipwreck located in the bay of Mindelo and at a maximum depth of 15 m; São Pedro, home to several green turtles saved by the community; Cubos, a scuba-diving hotspot; and Praia Grande Known as a surfing hotspot because of the huge waves of the “Casa de Pasto”. The maximum depth reached during all deployment tests was 20 m.

#### Deployment of the PlasPi TDM

**Fig. 10:**
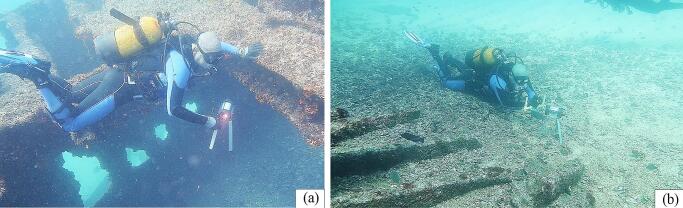
Deployment of the PlasPi TDM deployed on the São Macario shipwreck: (a) PlasPi TDM on the São Macario Shipwreck in the water column; (b) PlasPi TDM posed on the seafloor by a diver.

During the field tests, two types of deployment were made: Profiling and long-term Benthic observation. The profile deployment consisted of lowering the device into the water column. For this deployment mode, the data were collected during the descent of the device (Fig. 10 (a)). The data set was acquired at the rate of one image every 5 s, while temperature and pressure data were recorded every 10 s. The deployment for long-term benthic observation was done in most cases by diving, as shown in Fig. 10, and consisted of placing the device on the seafloor for 24 h (Fig. 10 (b)) and using an additional 8 kg weight to fix it firmly in the sediments.

Examples of test results demonstrating the capabilities of PlasPi TDM are shown in sections 7.3.2, 7.3.3 and 7.3.4.

#### Characterization of the marine environment

**Fig. 11:**
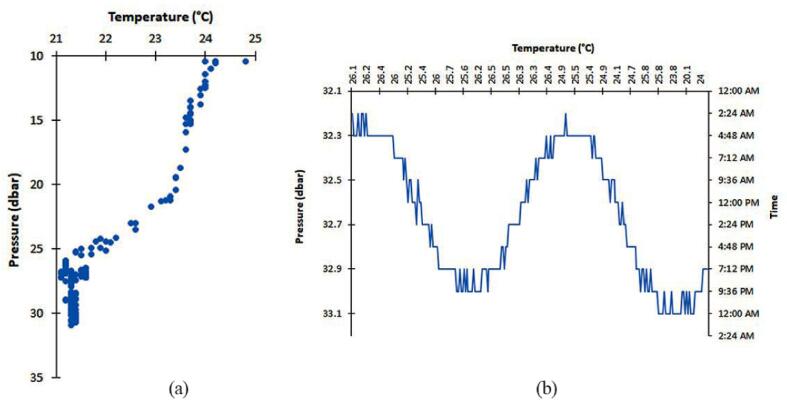
Temperature variation as a function of pressure (descend) during a field test at Cubos: (a) Profiling deployment; (b) 24 h deployment; At each measurement point, pictures, as well as multispectral data, were recorded.

The characterization of the marine environment was illustrated by analyzing the temperature and pressure data measured by the different sensors in profile deployment (Fig. 11 (a)) on 12/08/2021 and during the long-term benthic deployment (24 h) on the seafloor (Fig. 11 (b)) at Cubos on 18/10/2021. All deployments were by diving, and the maximum depth reached for both profile and Seafloor was 20.5 and 22.7 m, respectively.

##### Results of the profile and long-term benthic observation deployment

The temperature–pressure curve obtained from the profile deployment shows, expectedly, that temperature decreases with increased pressure. The average temperature value observed was 21.97 °C with a standard deviation of 1.02 °C. Regarding the long-term benthic monitoring (24 h), the device was deployed at a maximum depth of 22.78 m. The average bottom temperature during the 24-hour deployment was 25.49 °C with a standard deviation of 1.21 °C. Tidal patterns were also recorded including low tide during the day, and high tide, at night.

#### Bioassessment based on the images from the field tests and their annotation

**Fig. 12:**
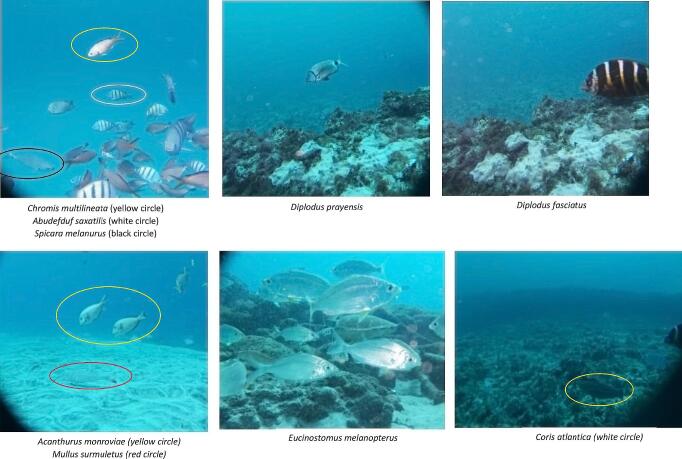

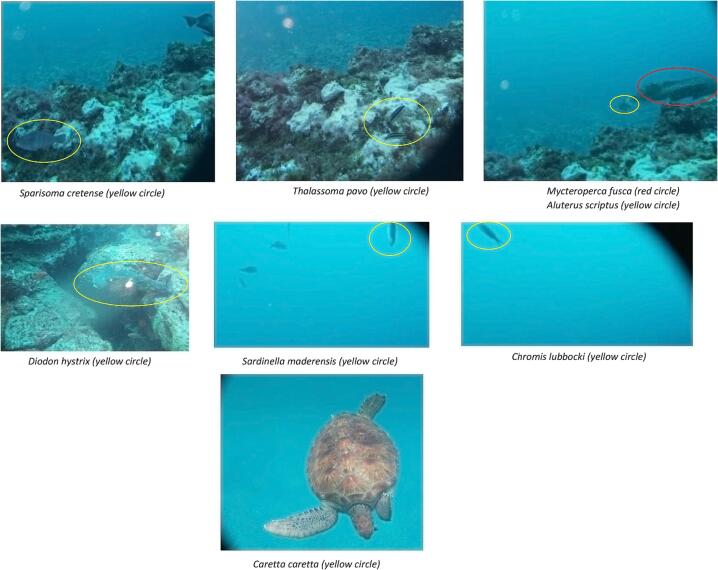
Some images of species taken by the PlasPi TDM during the test deployment.

**Fig. 13:**
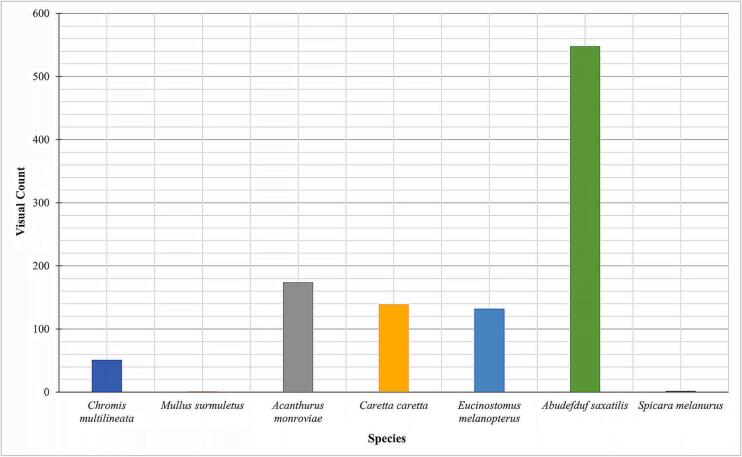
Visual counts of species from image annotation at São Pedro.

**Fig. 14:**
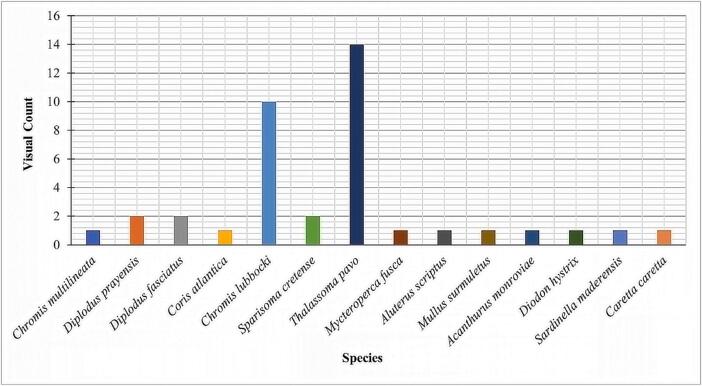
Visual counts of species from image annotation at Praia Grande.

Fig. 13 shows some of the images obtained from the field test deployments and made available in the BIIGLE online (https://biigle.de/) [27] platform for browsing and annotation. The image annotation allowed a complete interpretation of the different contents of an image revealing the significance of the PlasPi TDM in marine ecological and biological research. Image annotation in BIIGLE is based on a machine-learning algorithm consisting of a general object detection system that acquires knowledge of the structural features of objects of interest (in this case, different species) and non-interesting patterns from a set of image patches showing representative examples of all species [27]. For this paper, only the pictures obtained from São Pedro and Praia Grande were annotated with information and metadata attributes. The decision to analyze these two sites was based on the volume of data collected and the representativeness of species captured in the images. By focusing on this subset of sites, we were able to gather a significant amount of species data, enabling a more comprehensive analysis aligned with our research objectives. We annotated 197 images from which seventeen species, including two species endemic to Cabo Verde. We then grouped the species into 12 families (Table 1) noting the sites at which different species were observed (Fig. 12). The image annotation report (Fig. 13, Fig. 14) lists the occurrence of species appearing in each image. The most abundant species annotated were observed in São Pedro (Fig. 13), including *Abudefduf saxatilis* (548 individuals), *Acanthurus monrovia* (174 individuals), *Caretta caretta* (139 individuals), and *Eucinostomus melanopterus* (132 individuals). In terms of diversity, Praia Grande has a higher diversity with 14 different species (Fig. 14) against 7 species for Sao Pedro.

**Table 1:** Species identified from image annotation.

Types	Family	Species
Fish	Pomacentridae	*Abudefduf saxatilis*
		*Chromis multilineata*
		*Chromis lubbocki*
	Mullidae	*Mullus surmuletus*
	Acanthuridae	*Acanthurus monroviae*
	Gerreidae	*Eucinostomus melanopterus*
	Centracanthidae	*Spicara melanurus*
	Sparidae	Diplodus fasciatus*
		Diplodus prayensis*
	Labridae	*Coris atlantica*
		*Sparisoma cretense*
		*Thalassoma pavo*
	Serranidae	*Mycteroperca fusca*
	Monacanthidae	*Aluterus scriptus*
	Diodontidae	*Diodon hystrix*
	Clupeidae	*Sardinella maderensis*
Turtle	Cheloniidae	*Caretta caretta*

* Endemic species of Cabo Verde.

We also noted the data file sizes obtained during the 24-hour test. The environmental data files including temperature and depth/pressure readings measured 11.2 KB. The spectral data file size measured 37.3 KB. Both the environmental and spectral data files were saved in CSV format while the pictures were saved in jpeg format. Regarding the image file size, Sao Pedro, the location with the largest number of pictures (3 3 1) taken, had a total image file size of 69.1 MB. This is an indication of the amount of data generated by the PlasPi TDM for each sensor. The data size would however vary depending on the specific parameters being measured and the duration of data collection.

#### Reflectance spectrum analysis

**Fig. 15:**
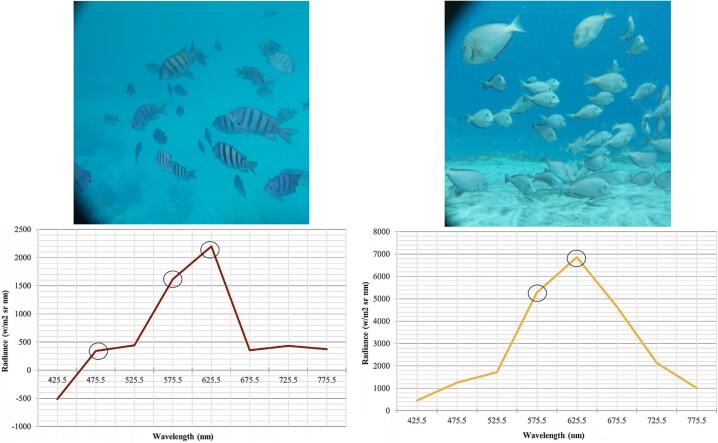
Spectral profiles of (a) *Abudefduf saxatilis*; (b) *Acanthurus monroviae* at 10 m depth. The circles show the peaks of wavelengths used for identification.

**Fig. 16:**
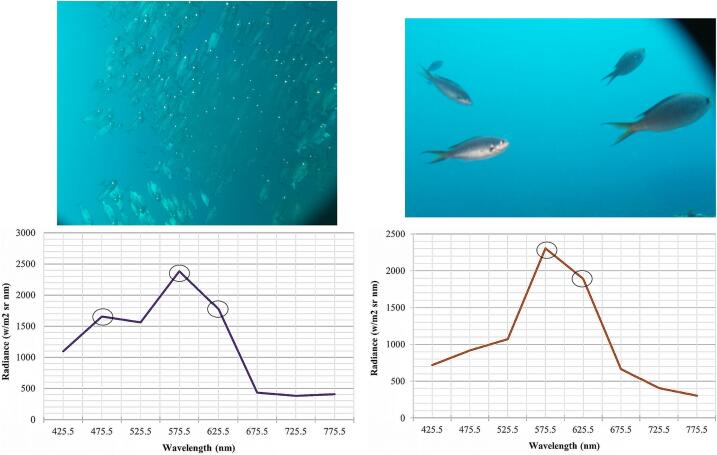
Different spectral profiles of species in deep-water at 25 m depth: (a) *Priacanthus arenatus*; (b) *Chromis multilineata*. The circles show the peaks of wavelengths used for identification.

The ultimate advantage of the PlasPi TDM is its added functionality enabling it to combine both photographic and spectral data acquisition for a more thorough identification of species and the monitoring of marine environmental conditions. The capture of the reflectance spectrum signatures of different species by the PlasPi TDM was demonstrated by applying the background reflectance spectrum correction. This approach excludes the non-specific reflectance spectrum (spectral background) superimposed on the object reflectance spectrum signal to obtain the net object reflectance signal of the image. Four spectral profiles were obtained from shallow water (10 m depth, Fig. 15) and deep water (25 m depth, Fig. 16) for four different species including *Abudefduf saxatilis*, *Acanthurus monroviae*, *Priacanthus arenatus*, and *Chromis multilineata*. A high reflectance of the species *Abudefduf saxatilis* and *Acanthurus monroviae* were recorded at 10 m depth in the wavelength 625.5 nm (Fig. 15, (a) and (b)).

To differentiate between species using the wavelength, the second and third-highest reflectances were registered. The second reflectance of *Abudefduf saxatilis* (Fig. 15 (a)) and *Acanthurus monroviae* (Fig. 15 (b)) occurred at 575.5 and 475.5 nm, and 575.5 nm, respectively. In contrast, the species *Priacanthus arenatus* (Fig. 16 (a)) and *Chromis multilineata* (Fig. 16 (b)), photographed in deeper waters (25 m depth) recorded their highest reflectance values in the 575.5 nm wavelength. The differentiating wavelengths for these species are 475.5 and 625.5 nm in the school of *Priacanthus arenatus*, while the wavelength for the *Chromis multilineata* was only 625.5 nm. These results reveal that the wavelengths facilitate species identification as well as indicate the section of the water column in which the image was taken. This is because the peak wavelength observed at 10 m depth is longer (625.5 nm), and hence has a shorter frequency and lower energy compared to the 25 m depth (575.5 nm), where the frequency and energy are higher. The differences in reflectance spectra observed at the species level were due to their unique optical properties, which depend on their physical, biological, and chemical characteristics. Moreover, differences in energy absorption coupled with the anatomical and morphological differences in species, enhancing light scattering at different wavelengths to permit taxonomic differentiation [28], could also account for the observed differences in reflectance spectra.

### Discussion and conclusion

This study presents a new low-cost, automatically operating research tool for continuous advanced short and long-term underwater ecological observations. The objective of this work was to upgrade the PlasPi marine camera designed by Purser et al., 2020 by adding new functionalities/sensors that record temperature and pressure, and multispectral sensors. The goal was to design a low-cost oceanographic instrument capable of measuring both the biotic and physical properties of seawater and make it available, and accessible to the coastal, resource management, and scientific communities in Africa and other low-income regions.

The PlasPi TDM has the capabilities to support marine ecology, biology, and physical oceanographic field studies. Its image and video capture capabilities allow for the extraction of valuable data, particularly regarding species occurrence, which plays a vital role in addressing research questions and hypotheses for the rapid assessment, monitoring and conservation of the marine environment and its biodiversity. To ensure optimal efficiency and compatibility of the PlasPi TDM with current image treatment techniques and Convolutional Neural Network (CNN) protocols for species classification and tracking, it is essential to consider specific data format, storage, and processing requirements. Firstly, the PlasPi TDM already satisfies the requirement of providing image data in JPEG format, which is commonly used in deep learning applications. Additionally, attention should be given to the size and resolution of the images, as resizing or cropping them to match the desired input dimensions of the model may significantly impact the accuracy [33]. It is important to note that the processing of image data, including manual annotations using platforms like BIIGLE, can be a time-consuming task. Therefore, preprocessing the image data becomes crucial to enhance its quality and improve the performance of the CNN model [34]. Hence, by carefully adhering to these considerations, researchers can ensure seamless integration of the PlasPi TDM image outputs into automated image analysis workflows, minimizing manual processing efforts and allowing for more efficient species identification and tracking.

In light of Howell et al.'s (2010) [35] findings regarding the application of camera systems in deepwater environments for habitat mapping and marine protected area site designation, as well as the insights provided by Bicknell et al., (2016) [13], the potential of the PlasPi TDM becomes even more significant. The PlasPi TDM, with its ability to capture detailed imagery of coastal ecosystems, including sediment composition, fauna, and flora, shares a similar objective with the camera systems used in deepwater environments. By leveraging the PlasPi TDM's capabilities in systematic and regular surveys, it can assist in the broader goal of habitat mapping and marine protected area site designation in coastal areas.

Furthermore, the PlasPi TDM holds potential for marine pollution monitoring efforts. Nowadays, coastal areas are increasingly affected by marine pollution originating from various nonpoint and point sources which has detrimental consequences on the environment, leading to eutrophication, loss of biodiversity, and disruptions to vital ecosystem functions [36]. The PlasPi TDM's capability to provide a synoptic view through its imagery and video functions allows for effective monitoring and assessment of marine pollution. By capturing visual evidence of algal blooms, sediment contamination, and other indicators of pollution, including oil spills, the PlasPi TDM can contribute to a better understanding of the extent and impacts of marine pollution in coastal ecosystems. This information is crucial for developing targeted mitigation strategies and implementing effective environmental management measures.

PlasPi TDM also observes changes in physical oceanographic properties including temperature and pressure, in relation to observed ecological data. Indeed, climate change alters the physical properties of the oceans leading to changes in heat transport and ocean circulation that impact the climate system. Therefore, measuring changing physical properties of the ocean is essential to understand the coastal and ocean dynamics and vulnerabilities at different temporal and spatial scales.

The integration of photographic and spectral data represents a novel approach that facilitates the identification and classification of marine organisms based on their reflectance peaks in different wavelengths. The spectral data adds another source of information that could support the classification of species in addition to classical methods. Ensuring the effective recognition of specimens and enabling cross-validation requires the implementation of appropriate data storage and organizational strategies. By designing a robust database structure and standardized protocols for data formatting and annotation, spectral reflectance measurements and corresponding object classification outputs can be efficiently stored and retrieved. This could involve creating a centralized database where spectral data and associated metadata (e.g., species information, collection location, date) are stored. Additionally, establishing standardized protocols for data formatting and annotation can facilitate data sharing and integration across multiple research projects. To establish accurate classification ranges or thresholds, data-driven approaches like machine learning algorithms can be employed. These algorithms analyze known species' spectral reflectance patterns and develop models to assign unknown specimens to specific species based on their spectral signatures. Defining criteria or thresholds that differentiate between taxa or species minimizes the chances of misclassification.

### Future directions

The PlasPi TDM is part of a growing trend in the development of cost-effective camera systems for marine research, particularly benefiting under-researched regions like Africa. Several previous studies have explored similar approaches, utilizing technologies such as stereo cameras and Raspberry Pi-based systems for underwater imaging [37], [38], [39]. These camera systems have been utilized for tasks such as 3D object detection, fish abundance assessments, and 3D reconstruction of the underwater environment. These examples highlight the increasing importance of camera systems in marine research, enabling the assessment of animal behaviour, fish abundance, and the reconstruction of underwater environments. The PlasPi TDM aligns with this trajectory, offering a versatile and cost-effective solution for advanced underwater observations. By leveraging the insights and experiences gained from previous camera systems, the PlasPi TDM contributes to the broader understanding of marine ecosystems and supports ecological monitoring and conservation efforts. Integrating the PlasPi TDM with established mobile platforms (CTD, Profilers, etc.) and robotic technologies (AUV, ROV, etc.) enhances its potential, unlocking new perspectives and comprehensive data for advancing our understanding of marine habitats and biodiversity. In the absence of expansive platforms, the PlasPi TDM has the potential to unlock new perspectives and provide comprehensive data for advancing our understanding of marine habitats and biodiversity. While these platforms have drawbacks, such as requiring fully-crewed ships and high costs, the monitoring of marine-coastal environments can benefit from the use of more modular, compact, and low-cost technologies [40], [41], [42], [43], [44] capable of performing frequent surveys in shallow water [45], [46]. The versatility and adaptability of the PlasPi TDM make it well-suited for various research needs, allowing for the exploration of diverse marine habitats and the investigation of the impacts of human activities and climate change on these ecosystems. By facilitating data collection and analysis in challenging marine environments, the PlasPi TDM opens up new frontiers of research, particularly in under-researched regions like Africa. By facilitating data collection and analysis in these areas, the PlasPi TDM contributes to filling critical knowledge gaps and broadens our understanding of global marine biodiversity.

Despite the many advantages of the PlasPi TDM, there are still several avenues to improve this observational system and enable it to reach its full potential. In the context of the PlasPi TDM's lighting system, further investigation is necessary to enhance its performance in challenging deep-sea environments. The effectiveness of LED lighting at great depths can be influenced by factors such as LED power and spectral quality. While the lighting system may provide satisfactory illumination in continental margin areas, it may encounter limitations when illuminating distant objects in the deep sea. Considering the diverse conditions encountered in different marine environments, including variations in depth and visibility, ongoing efforts to optimize LED power and spectral quality are crucial to improve illumination capabilities and ensure optimal performance of the PlasPi TDM. Furthermore, the camera's resolution could be improved by opting for a 4 k resolution camera. As for the watertight enclosure, future improvements of the prototype would increase the diameter or dimensions of the front cap and, therefore, of all the parts forming the waterproof enclosure. This will allow the different components of the front cap, mainly the camera, to be securely fixed inside to avoid image distortions. Similarly, the watertight enclosure should be equipped with a Pressure Relief Valve (PRV) to automatically release any excess pressure built up inside the watertight enclosure. It is also important to complement the proposed photographic and spectral integration approach with DNA-based methods, which could be utilized for future data validation and ground-truthing purposes.

The future improvements will lead to an improved underwater observational system enabling the PlasPi TDM to be used as a systematic observational tool across marine institutions in Africa and other countries. This tool therefore has the potential to enhance our understanding of the marine environment, and the changes therein, especially in the contemporary context of accelerated climate and anthropogenic changes. In addition, a rich database of species occurrences, their spectral signatures, and ancillary information on their habitat conditions will be established from the data collected. Finally, elaborate field trials near the coast will be integrated into regular coastal monitoring efforts to document changes in biodiversity across Africa.

## Declaration of Competing Interest

The authors declare that they have no known competing financial interests or personal relationships that could have appeared to influence the work reported in this paper.
